# Metabolic checkpoints in the regulation of Th17 cells: implications for uveitis pathogenesis and therapy

**DOI:** 10.3389/fimmu.2026.1690141

**Published:** 2026-02-10

**Authors:** YongFei Ma, QiuJin Zhang, ZhiXiang Ding

**Affiliations:** 1 The First Affiliated Hospital of Guilin Medical University, Guilin, China; 2 Department of Immunology, Guilin Medical University, Guilin, China

**Keywords:** metabolic checkpoint, metabolic reprogramming, Th17, Tregs, uveitis

## Abstract

The disruption of the balance between helper T cells (Th17) and regulatory T cells (Tregs) is associated with various autoimmune diseases. Th17 cells contribute to inflammation, whereas Tregs play a crucial role in suppressing autoimmunity. Th17 cells are central to the pathogenesis of autoimmune diseases, with specific metabolic pathways, enzymes, signaling pathways, and transcription factors acting as key checkpoints that influence T cell differentiation and immune responses. This review aims to summarize recent advances in understanding the role of Th17 cells and the Th17/Treg immune balance in the pathogenesis of uveitis, focusing on the impact of metabolic reprogramming on the activation, differentiation, and effector functions of T cells. Understanding the regulators of key metabolic checkpoints offers promising prospects for the treatment of autoimmune diseases, including Th17-mediated uveitis.

## 1 Introduction

Uveitis refers to intraocular inflammation of the uvea (including the iris, ciliary body, and choroid) and adjacent structures (including the retina and vitreous), caused by abnormal immune system activation. Anatomically, uveitis is classified into anterior, intermediate, posterior, and panuveitis (1). In terms of etiology, uveitis is categorized as either infectious or non-infectious, with non-infectious uveitis generally considered immune-mediated (2). Examples of non-infectious uveitis include acute anterior uveitis (AAU), as well as uveitis associated with Behçet’s disease (BD) and Vogt-Koyanagi-Harada disease (VKH) (3).

Uveitis is a leading cause of preventable blindness worldwide. Treatment primarily relies on local or systemic corticosteroids, often in combination with immunomodulatory drugs, to downregulate the immune response and reduce inflammation. However, current treatment methods are limited by side effects, including cataracts, glaucoma, and osteoporosis, as well as by a high recurrence rate and difficulty in achieving long-term remission. Therefore, there is an urgent need to develop more effective treatment strategies.

Helper T cell 17 (Th17) plays a central role in immune homeostasis, inflammatory responses, and autoimmune diseases, and is a key factor in the pathogenesis of uveitis (4). Th17 cells are characterized by the production of signature cytokines such as interleukin (IL)-17, IL-21, and IL-22, the expression of the major transcription factor retinoic acid receptor-related orphan nuclear receptor γT (RORγt), and the secretion of proinflammatory cytokines like granulocyte macrophage colony-stimulating factor (GM-CSF) and interferon-γ (IFN-γ), which contribute to ocular inflammation and tissue damage (5). In addition, Th17 cells can also secrete cytokines, including IL-10 and TGF-β, which suppress inflammation and modulate immune responses (6), thus exhibiting dual functions in the immune system.

A balance between Th17 cells and regulatory T cells (Tregs) is essential for immune homeostasis (7). There exists a functional antagonism between Th17 cells and Tregs (8), with the differentiation of these cells regulated by distinct transcription factors, RORγT for Th17 cells and Foxp3 for Tregs. Tregs primarily secrete cytokines such as IL-10 to exert immunosuppressive effects. The imbalance between Th17 cells and Tregs is a hallmark of autoimmune diseases, including uveitis (9). T cell differentiation and function are regulated through metabolic checkpoints (10). Unlike naïve T cells, effector T cells have high energy demands and rely on metabolic reprogramming, particularly aerobic glycolysis (11), to support their rapid proliferation and effector functions.

In this review, ‘metabolic checkpoints’ refer to metabolism related molecules or pathways that play a key regulatory role in the activation and differentiation of T cells. The differentiation, proliferation and effector functions of Th17 cells are carefully regulated by a series of metabolic checkpoints that integrate signal transduction and metabolic reprogramming, thereby precisely regulating immune cell fate (such as Th17/Treg balance), function and pathogenicity. These checkpoints include key metabolic enzymes (such as mTOR, AMPK, HIF-1 α), epigenetic regulators (such as sirtuins, HDACs), metabolic enzymes (such as lactate and succinate), etc. These checkpoints are closely related to metabolism, gene expression and immune cell fate.

This review will further investigate the metabolic checkpoints that regulate the function of Th17 cells, explore their impact on the pathogenesis of uveitis, examine the epigenetic modifications and metabolic reprogramming processes that influence the Th17/Treg balance, and provide a theoretical framework for identifying new metabolic targets and developing more effective therapeutic interventions for uveitis.

## 2 Differentiation, function, and pathogenic role of Th17 cells in uveitis

### 2.1 The role of RORγT in Th17 cell differentiation

Retinoic acid receptor-related orphan nuclear receptor gamma (RORγt) is a key transcription factor that regulates Th17 cell differentiation and cytokine production. It modulates Th17 differentiation by controlling IL-17 transcription and enhancing IL-17 production (12). The activity and expression of RORγt are regulated by various signaling pathways and transcription factors. Key regulatory mechanisms involve metabolic sensors and complex signaling cascades. For example, signal transducer and activator of transcription 3 (STAT3) regulating RORγt expression. Studies have shown that Th17 cytokine production relies on the RORγt-STAT3 signaling pathway (13).

Therapeutic strategies targeting RORγt typically involve small molecule inhibitors, including antagonists and inverse agonists, designed to block Th17 cell differentiation and function. For example, compounds CQMU151 and CQMU152 competitively bind to the endogenous ligands of RORγt, such as steroids (14), inhibiting Th17 lymphocyte subset differentiation, specifically reducing IL-17 production, and decreasing Th17 cell infiltration into the retina. In experimental autoimmune uveitis (EAU), matairesinol (MAT) exerts an immunosuppressive effect by inhibiting IRBP-specific Th17 cell differentiation and blocking MAPK and RORγt signaling (15). RORγt inhibitors reduce IL-17 protein expression in the retina of EAU mice, highlighting their potential as therapeutic targets for uveitis and other Th17-mediated autoimmune diseases.

### 2.2 Pro-inflammatory cytokines produced by Th17 cells and their effects

Interleukin-17 (IL-17) is the primary pro-inflammatory cytokine secreted by Th17 cells, and its expression is regulated by RORγt. Previous studies have demonstrated that IL-17A promotes neutrophil recruitment and the production of neutrophil extracellular traps (NETs) (16, 17), thereby exacerbating the inflammatory response in ocular tissue. In experimental autoimmune uveitis (EAU), neutralizing IL-17A significantly reduces NET formation, inhibits Th17 cell differentiation, and alleviates EAU symptoms (18). Disrupting the NETs-CD44-IL-17A feedback loop may serve as a potential therapeutic target for Behçet’s uveitis. Additionally, overexpression of Kallistatin promotes IL-17A release from IRBP-specific T cells, further exacerbating the inflammatory response in the EAU model (19). Interleukin-21 (IL-21) is another pro-inflammatory cytokine secreted by Th17 cells (20). In the experimental autoimmune uveitis (EAU) model, IL-21 promotes Th17 cell differentiation, and the absence of IL-21 signaling reduces IL-17 production and alleviates EAU symptoms in mice (21). Therefore, IL-21 is a key target that enhances Th17 activity while inhibiting Treg cells in autoimmune diseases (22). The role of interleukin-22 (IL-22) in uveitis remains controversial. A previous study using the experimental autoimmune uveitis (EAU) model demonstrated that IL-22 induces regulatory CD11b^+^ antigen-presenting cells (APCs) to convert pathogenic T cells into regulatory T cells (23), thereby protecting mice from uveitis. Subsequently, IL-22-producing CD4^+^ T cells were detected in the ocular specimens of patients with Behçet’s disease, and the concentrations of both IL-22 and TNF-α were significantly elevated, suggesting the pro-inflammatory role of IL-22 (24).

The effect of granulocyte-macrophage colony-stimulating factor (GM-CSF) on myeloid cell development is closely linked to the progression of inflammatory and autoimmune diseases (25). In the experimental autoimmune uveitis (EAU) model, mice developed inflammation even in the absence of IFN-γ and IL-17A, suggesting that GM-CSF activates eosinophils to promote ocular inflammation (26). Reducing GM-CSF secretion helps alleviate the enhancing effect of antigen-presenting cells (APCs) on Th17 cell pathogenicity (27).

In the pathogenesis of uveitis, IL-17, IL-21, and GM-CSF produced by Th17 cells are involved in the inflammatory response. A comprehensive understanding of these cytokines is crucial for developing targeted treatment strategies for uveitis.

### 2.3 Th17/Treg balance in uveitis

The regulation of Th17/Treg balance is of great significance in the pathogenesis and treatment of uveitis (28). Tregs play a key role in maintaining immune tolerance (29), with Foxp3 expression being a defining characteristic of these cells (30). Tregs secrete cytokines such as IL-10, TGF-β, and IL-35 (31). The differentiation of activated CD4^+^ T cells into Th17 or Treg cells depends on the balance between the lineage-specific transcription factors RORγT and Foxp3 (32).

Specific signaling and cellular metabolic pathways play a crucial role in T cell differentiation and the Th17/Treg balance. Signal transduction pathways, such as transforming growth factor-beta (TGF-β), promote the expression of Foxp3 by activating inhibitors of the Smad signaling pathway, thereby driving the differentiation of naïve T cells (Th0) into Tregs and suppressing inflammation and autoimmune responses (33, 34).

Cytokines play a pivotal role in maintaining the Th17/Treg balance. Interleukin-2 (IL-2) regulates the differentiation and function of Treg cells (35), promoting Treg formation and survival. Interleukin-27 (IL-27) inhibits Th17 differentiation and exerts an inhibitory effect in uveitis (36, 37). Interferon-gamma (IFN-γ) mediates the induction of IL-27 in target tissues, suppressing IL-17 responses, improving symptoms of experimental autoimmune uveitis in mice, and modulating the autoimmune response (38).

**Figure 1:**
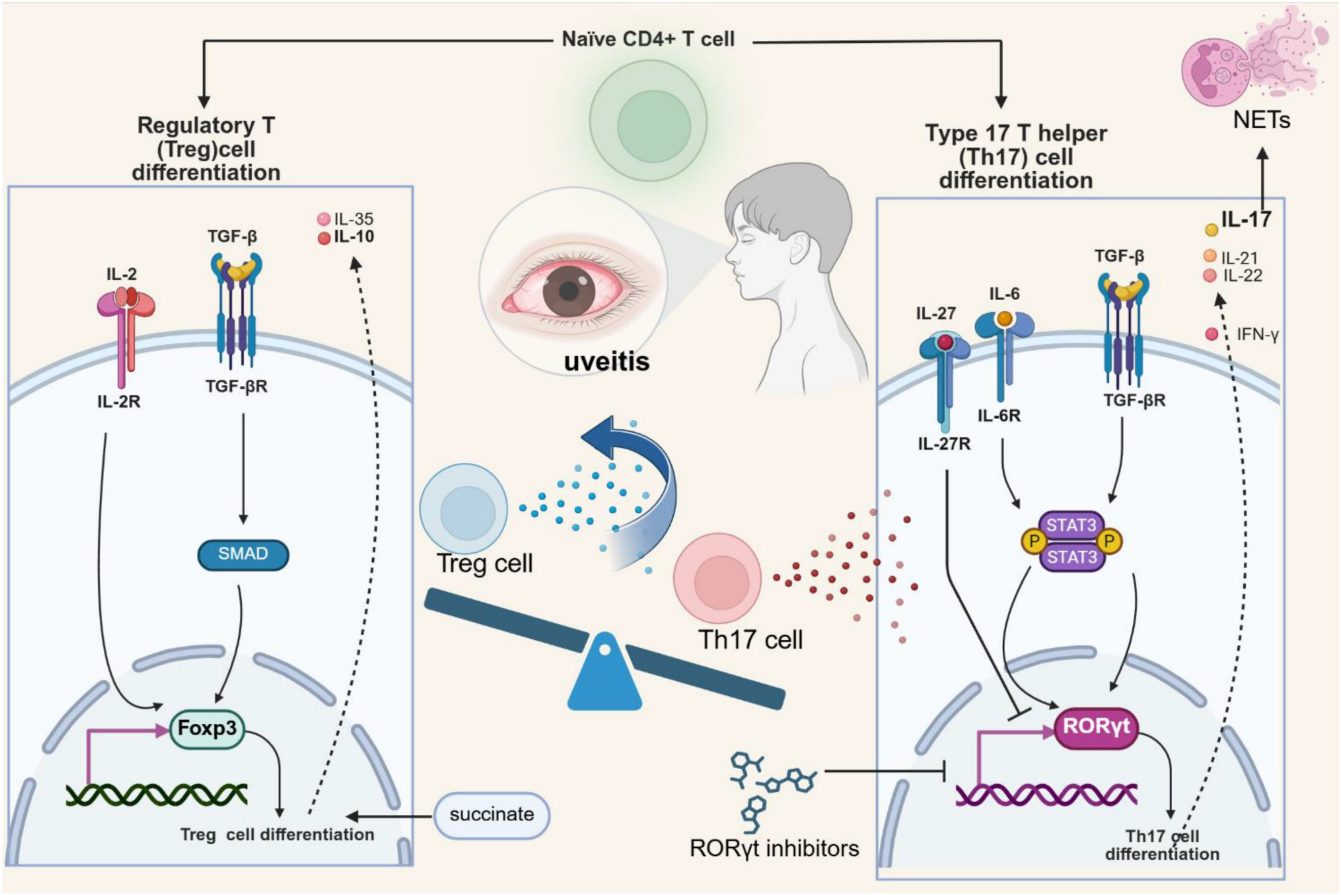
The pathogenesis of uveitis involves complex cytokine networks and signaling pathways. In particular, the differentiation of Th17 and Treg cells, along with the production of cytokines such as IL-17, IL-21, and IL-10, plays a pivotal role. An imbalance between Th17 and Treg cells is considered a key driver of uveitis.

IL-17A, a hallmark cytokine produced by Th17 cells, induces IL-23 expression through the NF- κ B signaling pathway, establishing an autocrine negative feedback mechanism (39). This induction subsequently limits the secretion of other pro-inflammatory Th17 related cytokines, including IL-17A and IL-17F, by upregulating inhibitory molecules such as SOCS proteins (40). This key regulatory pathway has also been identified in human Th17 cells, highlighting its relevance in the translation process. Recent evidence points to ubiquitin-specific peptidase 1 (USP1) as a critical orchestrator of Th17/Treg polarity, promoting Th17-cell differentiation and attenuating Treg-cell development through TAZ deubiquitination. A stabilized TAZ protein subsequently enhances the transcriptional activity of RORγt, and concurrently facilitates the proteasomal degradation of Foxp3 (41). This therapeutic potential is underscored by studies involving ML323, a specific inhibitor of the USP1/UAF1 deubiquitinase complex. These findings posit ML323 as a promising candidate for the treatment of diseases driven by a Th17/Treg imbalance, such as autoimmune and inflammatory conditions. Consequently, targeting the USP1-TAZ axis represents a novel strategic avenue for immunomodulatory therapy (**Figure 1**).

Specific metabolic enzymes also influence the Th17/Treg cell balance. Phosphoinositide 3 kinase (PI3K) plays an important role in regulating Th17/Treg homeostasis in autoimmune diseases through phosphorylation of PI3K/Akt/FoxO1 signaling pathway. Akt regulates T cell differentiation through the negative regulation of transcription factor forkhead box O1 (FoxO1). FoxO1 increases the expression of Foxp3 in CD4+T cells and enhances the number and ability of Treg cells (42). PIM1 kinase reduces the proportion of Th17 cells while increasing the proportion of Treg cells in experimental autoimmune uveitis (EAU) through the AKT/FOXO1 signaling pathway (43). The upregulation of PIM1 in CD4^+^ T cells in Vogt-Koyanagi-Harada (VKH) disease suggests that targeting PIM1 kinase may offer a potential therapeutic strategy for uveitis, particularly VKH (44). Additionally, progesterone (PRG) reduces the pathogenicity of Th17 cells in autoimmune uveitis via the ID2/PIM1 axis (45). Ornithine decarboxylase 1 (ODC1) inhibits STAT5A phosphorylation in CD4^+^ T cells, disrupting the Th17/Treg balance (46).

Given the central role of Th17/Treg imbalance in uveitis (47), treatment strategies aimed at restoring this balance hold significant promise. Actively exploring the regulation of the Th17/Treg axis through specific pathways or cytokine-based interventions may provide valuable insights for the treatment of uveitis (48).

## 3 Metabolic reprogramming in Th17 cell differentiation

Metabolic reprogramming is a hallmark of activated T cells and plays a crucial role in Th17 cell function and autoimmune pathogenesis (49). Activated T cells, including Th17 cells, undergo a profound metabolic shift from a state that is mainly dependent on oxidative phosphorylation (OXPHOS) to a state dominated by aerobic glycolysis. Meanwhile, fatty acid metabolism plays a key regulatory axis role in shaping the immune homeostasis of Th17 cell fate and regulatory T (Treg) cell fate. Amino acid metabolism, especially glutaminolysis and serine metabolism, is also crucial for the expansion and function of Th17 cells, directly affecting their ability to drive inflammation. This section explores the metabolic adaptations that occur during Th17 cell differentiation, focusing on the reliance on specific metabolic processes to meet the high energy and biosynthetic demands of activated T cells. It highlights the interactions between metabolic pathways, epigenetic regulation, and Th17 cell pathogenicity, emphasizing their collective impact on immune responses.

### 3.1 Glucose metabolism: glycolysis and oxidative phosphorylation

Glucose metabolism provides essential ATP for Th17 cell proliferation and cytokine production, influencing epigenetic modifications, inflammatory gene expression, and driving autoimmune responses (50). The upregulation of glucose transporters, such as GLUT1 and GLUT3, along with the production of the glycolytic byproduct lactate, plays a key role in regulating Th17 differentiation and autoimmune inflammation.

Enhanced glycolysis provides the necessary ATP and biosynthetic intermediates for the rapid proliferation and cytokine production characteristic of Th17 cells. Phosphoenolpyruvate (PEP), an intermediate of glycolysis, is particularly important as it maintains cytosolic calcium levels by suppressing sacro/endoplasmic reticulum calcium ATPase activity. Increased PEP production has been shown to enhance T cell responses (51). Pyruvate kinase 2 (PKM2), a key enzyme in the final step of glycolysis, generates pyruvate and ATP, and has been implicated in histone modification by phosphorylating histone H3T11, thereby linking glucose metabolism to chromatin dynamics (52).

Activated T cells undergo metabolic reprogramming, whereas immature T cells primarily rely on oxidative phosphorylation (OXPHOS) for energy production, which supports Th17 differentiation and effector function (53). T cell activation involves a transition from a state dependent on oxidative phosphorylation (OXPHOS) to one dominated by aerobic glycolysis (54). This metabolic shift is essential for regulating cell function and promoting the proliferation and differentiation of Th17 cells (55). The balance between glycolysis and OXPHOS dictates the interaction between T cell function and energy metabolism.

This metabolic transition involves the upregulation of glucose transporters, such as glucose transporter 1 (GLUT1), which influences T cell activation and metabolic reprogramming (56, 57). GLUT1-dependent molecular switches promote glycolysis, whereas GLUT1 knockdown inhibits glycolysis and slows the transition of human T cells from a quiescent to a proliferative state (58). GLUT1 inhibitors, such as BAY876, transiently suppress autoreactive T cells by restricting glucose uptake via the GLUT1 transporter (59). In addition to GLUT 1, glucose transporter 3 (GLUT 3)-dependent glucose uptake regulates the metabolic transcriptional circuitry of Th17 cells and controls their pathogenicity through epigenetic reprogramming (60). Inhibiting the Glut3-dependent acetyl-CoA metabolic checkpoint may alleviate Th17 cell-mediated inflammatory diseases.

**Figure 2:**
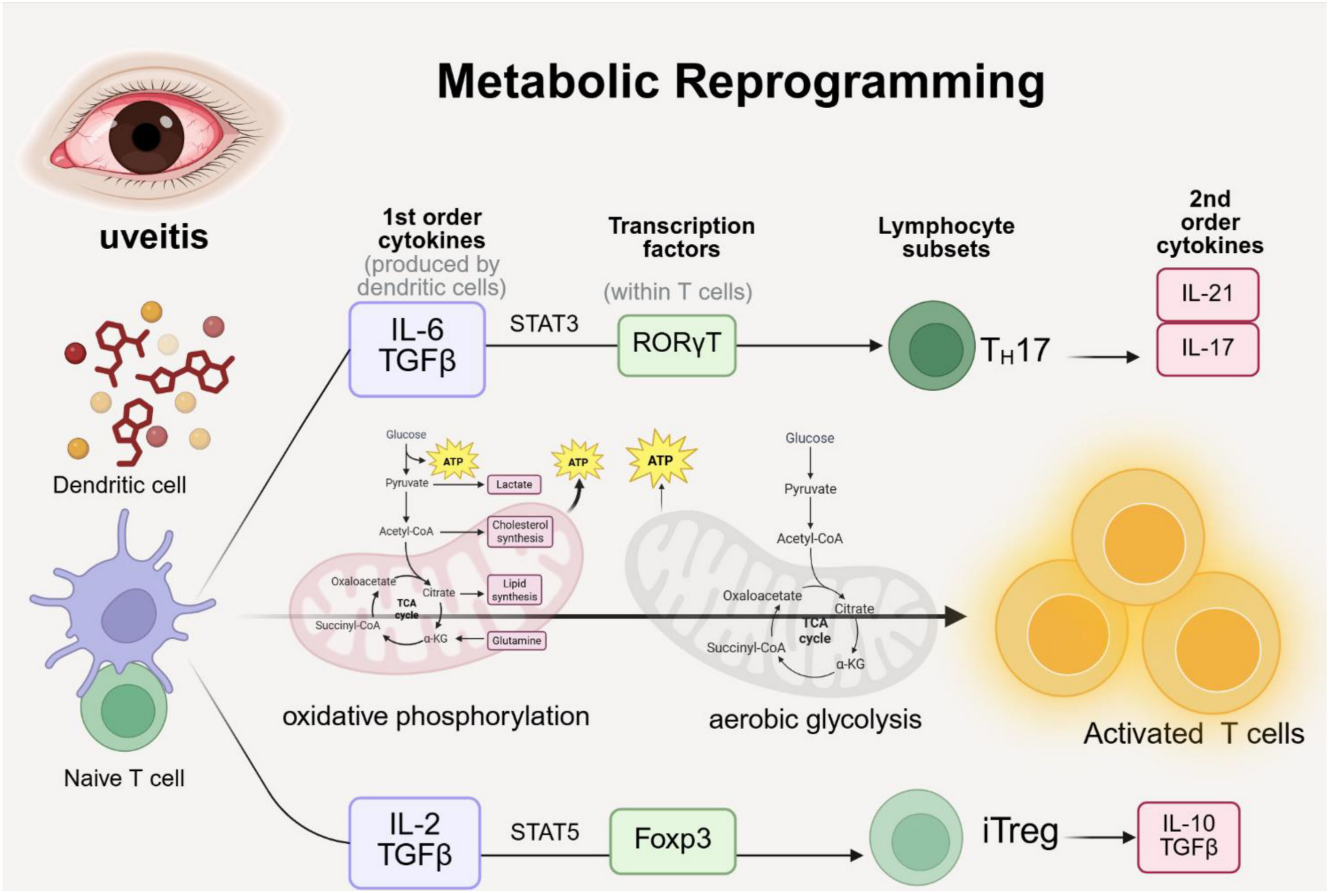
Metabolic reprogramming in uveitis requires both oxidative phosphorylation and aerobic glycolysis to support the transition from immature to activated T cells. Upon activation, T cells differentiate toward pro-inflammatory Th17 cells or immunosuppressive Treg cells, thereby influencing disease progression.

Additionally, Th17 cells rely on aerobic glycolysis to meet their high metabolic energy demands, and the accumulation of lactate limits T cell responses. For instance, dichloroacetic acid (DCA) inhibits glycolysis through metabolic reprogramming, reducing lactate levels in CD4^+^ T cells and highlighting the role of glycolysis in autoimmune inflammation (61) (**Figure 2**).

### 3.2 Fatty acid metabolism in immune cells

The regulatory role of fatty acid metabolism in Th17 and Treg cell differentiation is a key pathological mechanism in inflammation and autoimmune diseases (62). The development of Th17 cells is strongly dependent on *de novo* fatty acid synthesis (FAS), while Treg cell differentiation and function are closely linked to fatty acid oxidation (FAO). Thus, fatty acid synthesis and its regulation significantly influence Th17 cell differentiation and the counter-regulatory effects of Treg cells (63).

Th17 cell development is highly dependent on *de novo* fatty acid synthesis (FAS). Key enzymes involved in this process include Acetyl-CoA carboxylase 1 (ACC1) and Fatty Acid Synthase (FASN), which are crucial for the synthesis of long-chain fatty acids (LCFAs). In contrast to Th17 cells, Treg cell differentiation and function are significantly supported by fatty acid oxidation (FAO). FAO provides energy through mitochondrial respiration, which is crucial for activating CD4+ T cells and subsequently promoting Treg cell differentiation. For instance, carnitine palmitoyltransferase 1A (CPT1A) is identified as the rate-limiting enzyme of FAO, which promoting the inflammatory response (64).

The opposing metabolic demands of Th17 and Treg cells constitute a key regulatory axis. Inhibition of FAS or promotion of FAO in fatty acid metabolism can alter this balance, driving the differentiation of Th17 cells toward Treg cells and shifting the axis toward Treg-mediated immunosuppression. Inhibition of acetyl-CoA carboxylase 1 (ACCA1), a key enzyme in *de novo* fatty acid synthesis, promotes Treg cell differentiation while inhibiting Th17 cell differentiation (65), identifying ACC1 as a metabolic checkpoint for Treg cells. Experiments using etomoxir, an inhibitor of the rate-limiting enzyme in long-chain fatty acid oxidation (LC-FAO), have demonstrated that metabolic pathways other than LC-FAO can also promote Treg differentiation (66). Short-chain fatty acids (SCFAs), such as butyric acid, enhance stable Foxp3 expression and Treg cell differentiation (67), thereby influencing inflammatory responses in various organs.

### 3.3 Amino acid metabolism in immune cell function

Amino acid metabolism plays a crucial immunomodulatory role in inflammatory responses and autoimmune diseases. Th17 cells exhibit a high metabolic demand, particularly for glutamine and serine, to support their proliferation and effector functions. Changes in the local concentration or metabolic utilization of these amino acids within the ocular microenvironment can modulate the pathogenicity of Th17 cells. Therapeutic strategies that restrict the availability of essential amino acids or inhibit their metabolic pathways may be effective in treating uveitis.

Glutamine metabolism plays a critical role in Th17 cell proliferation and cytokine production (68). It also regulates CD4^+^ T cell differentiation, promoting Th17 development while inhibiting Th1 differentiation (69). Amino acid transporters, such as LAT1 (encoded by SLC7A5) and ASCT2, are positively correlated with Th17 cell differentiation (70). Therefore, alterations in glutamine metabolism contribute to the pathogenesis of autoimmune diseases.

Serine metabolism influences the differentiation of Th17 cells by providing essential one-carbon units and precursors for nucleotide biosynthesis. This metabolic pathway is significantly upregulated in activated T cells. Inhibition of amino acid synthesis with aminoxyacetic acid (AOA) disrupts serine metabolism and reduces inflammation-associated antimicrobial peptides and Th17-related cytokines in a psoriasis mouse model (71). Furthermore, serine and glycine supplementation increases the expression of IL-17-mediated inflammatory genes, indicating a link between serine metabolism and Th17-driven inflammatory responses.

## 4 Key metabolic checkpoints regulating Th17 cell fate

Th17 cell function is governed by a series of metabolic checkpoints. This section examines the metabolic processes essential for Th17 pathogenicity, coordinated by regulators such as the mammalian target of rapamycin (mTOR), which orchestrates the metabolic transition from oxidative phosphorylation to aerobic glycolysis and *de novo* fatty acid synthesis. Epigenetic mechanisms tightly couple cellular metabolism with gene expression and immune cell fate, collectively influencing the balance between Th17 and regulatory T (Treg) cells. Elucidating these metabolic checkpoints and their upstream regulatory pathways may reveal novel therapeutic targets for autoimmune diseases, including Th17-mediated uveitis.

### 4.1 mTOR signaling

Mammalian target of rapamycin (mTOR) signaling plays a pivotal role in promoting Th17 cell differentiation. mTOR signaling functions as a key metabolic checkpoint, integrating immune receptor signaling with metabolic programs (72). The metabolic reprogramming driven by mTOR supports the high biosynthetic demands of Th17 cell differentiation, promoting the expression of transcription factors such as RORγt and cytokines such as IL-17 to coordinate immune responses (73).

Upon activation, mTOR drives the metabolic reprogramming of T cells, particularly by enhancing aerobic glycolysis and fatty acid synthesis (FAS), which together facilitate the differentiation of immature T cells into Th17 cells. The mTOR pathway comprises two distinct complexes, mTORC1 and mTORC2. Activation of mTORC1 influences lipid biosynthesis by inducing the expression of cholesterol-related genes through sterol regulatory element-binding proteins (SREBPs) (74).

The activity of mTOR is tightly regulated by diverse upstream signals and metabolites, and it plays a central role in maintaining the balance between Th17 and regulatory T (Treg) cells (75). mTOR activation promotes Th17 differentiation and glycolysis, whereas its inhibition enhances fatty acid oxidation (FAO) and supports Treg differentiation. The key transcription factor Foxp3, which defines Treg cells, can also inhibit their function by suppressing the PI3K–Akt–mTORC1 signaling pathway (76). Rapamycin inhibits the mTOR pathway, promotes Treg proliferation, modulates their function, and supports the maintenance of peripheral tolerance (77). Overall, mTOR signaling influences Th17 differentiation and cytokine production, making it a critical target for modulating T cell–mediated immune responses.

### 4.2 HIF-1α signaling

Hypoxia-inducible factor-1α (HIF-1α) enhances Th17 development. HIF-1α is a regulatory factor and metabolic sensor that promotes Th17 differentiation while inhibiting Treg cell development (78). Functioning downstream of mTOR, HIF-1α modulates the expression of key glycolytic enzymes. Further investigation is warranted to elucidate how HIF-1α–driven Th17 differentiation and glycolytic metabolism influence the balance between Th17 and Treg cells.

The mechanism by which HIF-1α promotes Th17 cell differentiation involves both direct interaction and transcriptional regulation. HIF-1α enhances Th17 development and modulates IL-17 production by directly activating RORγt transcription. By binding with RORγt at the IL-17a promoter, HIF-1α recruits the transcriptional co-activator p300, thereby facilitating Th17 cell differentiation and driving the transcription of Th17-specific genes (79). HIF-1α directly interacts with Foxp3 and promotes its degradation, thereby inhibiting Treg cell development (79). This reciprocal regulation of Th17 and Treg cells by HIF-1α is critical for maintaining immune homeostasis.

In addition to its role in transcriptional regulation, HIF-1α influences Th17 cell metabolism by inducing glycolytic enzymes and promoting glycolysis. For instance, HIF-1α upregulates the transcription of pyruvate dehydrogenase kinase 1 (PDK1), thereby enhancing aerobic glycolysis and supplying energy and biosynthetic precursors for the rapid expansion of Th17 cells (80). HIF-1α functions downstream of the mTOR pathway, integrating with metabolic reprogramming to drive lineage-specific differentiation. The Sirtuin 1–mTOR–HIF-1α axis exemplifies how upstream metabolic regulators influence HIF-1α activity and, consequently, T cell differentiation (81). AdipoR1 regulates Th17 cell differentiation through HIF-1α–dependent glycolysis (82). Targeting AdipoR1 with specific agonists or antagonists may offer novel strategies to suppress pathogenic Th17 responses and alleviate ocular inflammation, as well as to explore its therapeutic potential in uveitis and other autoimmune diseases (83).

### 4.3 Ribosomal protein S6 kinase beta-1

S6K1 is a serine/threonine kinase that phosphorylates ribosomal protein S6, thereby promoting protein synthesis through the regulation of mRNA translation initiation and elongation (84). As a key downstream effector of mTOR signaling, S6K1 modulates diverse inflammatory responses and cellular metabolic processes (85).

S6K1 plays a pivotal role in Th17 cell differentiation and IL-17 expression. Upon activation by mTORC1, phosphorylated S6K1 induces the early growth response gene 2 (EGR2), which promotes IL-17 production. EGR2 suppresses the expression of the growth factor independent 1 (GFI1) gene by directly binding to its promoter. In turn, GFI1 prevents RORγt recruitment to the IL-17 promoter, thereby reducing IL-17 expression (86). The S6K1-specific inhibitor PF-4708671 downregulates IL-17 expression by blocking the mTORC1–S6K1–EGR2 axis (87). Thus, targeting S6K1 provides a critical checkpoint for regulating Th17 cell fate and maintaining immune homeostasis.

### 4.4 Sirtuins

Sirtuins are nicotinamide adenine dinucleotide (NAD^+^)–dependent deacetylases that preserve protein function and stability through deacetylation. They are involved in diverse biological processes, including genome stability, cellular metabolism, and inflammatory responses (88), and play a pivotal role in immune regulation and metabolic homeostasis.

The seven mammalian sirtuins (Sirt1–Sirt7) display distinct subcellular localizations and specialized functions that support metabolic regulation in T cells (89). For instance, Sirtuin 1 promotes Th17 cell generation and function by enhancing RORγt transcriptional activity through deacetylation, thereby facilitating Th17 differentiation (90). Sirtuin 2 modulates IL-17A and IL-2 transcription, and its deacetylation activity activates the mTORC1/HIF-1α/RORγt pathway, driving Th17 cell differentiation (91).

Regulation of sirtuin activity in T cells represents a promising therapeutic strategy for inhibiting Th17 cell differentiation in autoimmune diseases. Modulating sirtuin function may restore the metabolic balance of Th17 cells, thereby influencing their differentiation, cytokine production, and the progression of uveitis.

### 4.5 HDACs

Histone deacetylases (HDACs) are a class of enzymes that regulate histone acetylation, working in conjunction with histone acetyltransferases (HATs) to modulate chromatin structure and gene expression. Elevated HDAC levels have been observed in peripheral blood mononuclear cells (PBMCs) of patients with autoimmune uveitis (AU), correlating with the differentiation of CD4^+^ effector T cells. HDACs regulate the CDK6/ID2 axis to form a positive feedback loop, thereby promoting the expression of PIM1 kinase, which enhances Th17 differentiation and pathogenicity, as well as the development of experimental autoimmune uveitis (EAU). Additionally, HDACs influence the Th17 cell cycle by deacetylating histone H3 and inhibiting the expression of p21 and p53 (92). Inhibition of HDACs can restore the Th17/Treg imbalance induced by experimental autoimmune uveitis (EAU), suppress Th17 differentiation and pathogenicity, and enhance the number of Treg cells, thereby re-establishing immune regulation. Thus, targeted HDAC inhibition holds significant therapeutic potential for reprogramming cell proportions, modulating the inflammatory response, and mitigating CD4^+^ T cell-mediated autoimmune processes (93), making it a promising strategy for treating uveitis.

### 4.6 AMPK signaling

Adenosine monophosphate-activated protein kinase (AMPK) activation inhibits Th17 cell differentiation, which is a central cellular energy sensor and regulator of T lymphocyte metabolism (94). AMPK activation promotes fatty acid oxidation, facilitating Treg cell development and immunosuppression. This metabolic reprogramming is characterized by enhanced fatty acid oxidation and the inhibition of *de novo* lipid synthesis.

**Figure 3:**
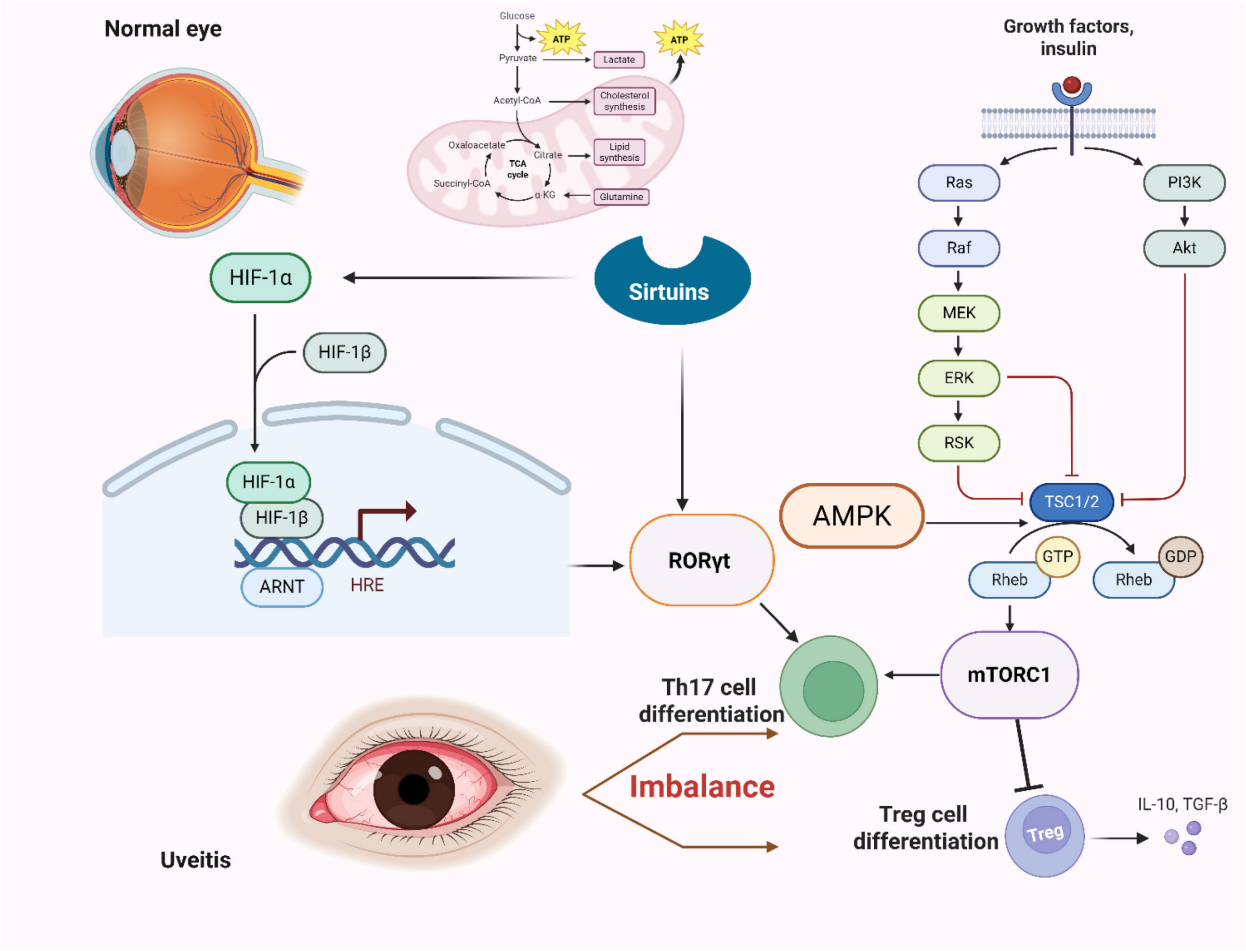
In the intraocular environment, it demonstrates the fate decision of naive T cells towards Th17 or Treg cell differentiation under the regulation of microenvironment signals and metabolic checkpoints. it also explores how key pathways such as glycolysis, fatty acid metabolism, mTOR, and HIF-1α are interrelated and influence differentiation. Important metabolic enzymes, sensors, and regulatory molecules are highlighted on the pathways, ultimately pointing to “Th17/Treg imbalance” and “uveitis pathology”.

The activation of AMPK, for instance, through the agonist AICAR, has been shown to inhibit Th17 cell induction *in vitro* (95, 96). This suggests that AMPK’s inhibitory effect on Th17 development is closely linked to metabolic regulation. Modulating AMPK activity can shift the balance of T cell subsets and alter the level of inflammation (**Figure 3**).

### 4.7 Other influencing factors

#### 4.7.1 Lactic acid modification

Lactate and its post-translational modifications play a significant role in the differentiation and gene expression of Th17 cells. Glycolysis regulates the site-specific lactation of proteins, which in turn promotes Th17 cell differentiation (97). Evidence from the experimental autoimmune uveitis (EAU) model highlights the importance of lactate in the Th17 cell-mediated immune response. A global lactate profiling study revealed that lactation of IKZF1 was significantly upregulated in CD4^+^ T cells from both normal mice and EAU animal models. Notably, high lactation at the Lys164 site of IKZF1 promoted Th17 differentiation by directly regulating the expression of Th17-related genes (98). This specific modification is strongly associated with IKZF1-mediated Th17 differentiation, suggesting that lactate modification could serve as a potential therapeutic target for autoimmune diseases.

#### 4.7.2 Succinate metabolism

Succinate metabolism plays a pivotal role in regulating the stability and function of Treg cells, thereby indirectly impacting the balance between Treg and Th17 cell populations. Studies have shown that succinate impairs Treg cell function and stability by reducing the expression of the key transcription factor Foxp3, thereby indirectly affecting the balance between Treg and Th17 cell populations. The reduction of succinyl‐CoA leads to reduced succinylation of Foxp3 on key lysine residues, especially K8 and K263 (99). The subsequent de‐succinylation exposes these lysine residues, rendering Foxp3 susceptible to ubiquitination and proteasomal degradation, impairing the function and stability of Treg cells.

#### 4.7.3 Hedgehog signaling

Hedgehog signaling selectively drives the polarization of Th17 cells (100). The endogenous hedgehog ligand, Indian hedgehog (IHH), transmits signals through the canonical pathway, involving the transcription factor Gli3, as well as through a noncanonical pathway that engages AMPK (101). The interaction between IHH, Gli3, and AMPK establishes hedgehog signaling as a metabolic checkpoint regulating this specific T cell lineage, offering a novel therapeutic strategy for Th17-mediated autoimmune diseases.

#### 4.7.4 Aromatic hydrocarbon receptor

The Aromatic Hydrocarbon Receptor (AHR) is a ligand-activated transcription factor that plays a crucial role in immune regulation. Kyne, an endogenous ligand of AHR, activates the receptor to promote the expression of Foxp3 in CD4^+^ T cells. This activation contributes to Treg cell differentiation and plays a critical role in maintaining immune tolerance (102). AHR activation by the ligand 2,3,7,8-tetrachlorodibenzo-p-dioxin (TCDD) significantly inhibits autoimmune uveoretinitis by promoting the expansion of CD25^+^ Foxp3^+^ Treg cells and suppressing the activation of Th1 and Th17 cells (103). Other AHR ligands, such as FIZC, have been shown to promote the generation of Th17 cells (104). These results suggest that AHR exerts dual effects on CD4^+^ T cell differentiation. Given that AHR regulates Th17 and Treg differentiation in a ligand-specific manner, selective activation or inhibition of the AHR pathway may help restore immune homeostasis.

### 4.8 Interaction between metabolism and post transcriptional regulation

Beyond direct regulation by metabolic enzymes and signaling pathways, the Th17/Treg balance is further fine-tuned by microRNAs (miRNAs), which serve as key post-transcriptional regulators. These miRNAs precisely target metabolic checkpoints and key transcription factors, adding an essential layer of immune control. In uveitis, specific miRNA profiles are dysregulated in patient peripheral blood and intraocular fluids, suggesting their potential as biomarkers for monitoring disease activity. Functional studies further substantiate the impact of individual miRNAs on immune homeostasis. For instance, plasma-derived exosomal miRNA-19b-3p is upregulated in patients with Beheet’s uveitis. This miRNA promotes Th17 cell differentiation while simultaneously suppressing Treg cell development. A proposed mechanism suggests that highly expressed miRNA-19b-3p induces Th17/Treg imbalance by downregulating CD46 expression (105). Similarly, the upregulation of miR-155 in experimental autoimmune uveitis models enhances the pathogenicity of Th17 cells, an effect mediated through cooperation with the transcription factor STAT3. Research in rheumatoid arthritis also confirms that miR-155 can influence the Th17/Treg balance by targeting key signaling molecules, supporting the role of such miRNAs as pivotal regulators in autoimmune pathology (106). Therefore, deciphering the intricate network of miRNA-metabolic axes offers a novel perspective for developing innovative therapeutic strategies. Targeting these upstream regulatory nodes could enable a more precise restoration of Th17/Treg immune homeostasis, presenting a promising approach for the treatment of uveitis and other autoimmune diseases.

## 5 Conclusion and prospect

This review provides a comprehensive analysis of the latest research on the metabolic checkpoints regulating Th17 cells in uveitis, highlighting the pivotal roles of metabolic checkpoints and reprogramming in determining Th17 cell differentiation and the pathogenesis of uveitis. Th17 cells act as key drivers of uveitis by secreting cytokines that significantly promote inflammatory responses and disease progression. Recent studies have elucidated the specific metabolic pathways and regulatory factors governing Th17 differentiation. Strategies aimed at balancing Th17 and Treg cell differentiation form the foundation of targeted therapeutic approaches for uveitis (107, 108).

Research on metabolic checkpoints provides a transformative perspective for the diagnosis and treatment of uveitis. The central innovation of this approach is its establishment of a direct link between immune cell function and intrinsic metabolic states, thereby bridging fundamental immunometabolism and clinical practice. By moving beyond a purely immunological viewpoint, it provides deeper mechanistic insights into autoimmune uveitis pathogenesis, centering on “immune metabolic reprogramming” as a key driver. Targeting these metabolic vulnerabilities lays a groundwork for developing safer, more effective therapies that extend beyond generalized immunosuppression.

While targeting metabolic checkpoints represents a promising therapeutic strategy for Th17 cell-mediated uveitis, its clinical translation encounters several significant challenges. First, core metabolic pathways like glycolysis are essential to the survival and function of nearly all nucleated cells. Systemically inhibiting these pathways may disrupt metabolic homeostasis in healthy tissues, leading to serious adverse effects—including excessive immunosuppression with increased infection risk or metabolic dysregulation that induces organ toxicity. Second, effective drug delivery to sites of ocular inflammation remains a major challenge, as systemic administration often fails to achieve therapeutic intraocular concentrations. The development of localized intraocular delivery systems, such as nanocarriers or hydrogels, represents a promising strategy to enable direct and sustained release of metabolic modulators to affected ocular tissues. This approach can enhance local efficacy while minimizing systemic exposure and related adverse events. Furthermore, combination therapies may help overcome resistance and reduce the toxicity associated with high-dose monotherapy. For instance, combining low-dose metabolic modulators (e.g., ACC1 inhibitors) with cytokine-targeting biologics (e.g., anti-IL-17A monoclonal antibodies) could synergistically restore Th17/Treg immune balance more effectively and safely.

Looking ahead, several key directions should be prioritized. First, it is essential to investigate the specific signal transduction processes of Th17 cells in the eye (109), particularly in light of the eye’s unique immune privilege, and to further explore the factors influencing Th17 metabolic reprogramming in diseases such as uveitis. Second, identifying novel metabolic targets beyond those currently known is critical, along with the development of more selective and effective metabolic interventions. How can drug design be optimized to address challenges related to selectivity, metabolism, and significant potential side effects? Could monoclonal antibody and RNA interference technologies be applied? Is there a need for combination therapies that employ different mechanisms of action? Third, promising preclinical findings should be translated into clinical applications, with rigorous preclinical and clinical trials used to evaluate the safety and efficacy of targeted therapeutic pathways.

The potential of targeting the metabolic pathway in Th17 cells as a novel therapeutic strategy for uveitis is significant (110). Current biological agents and transcription factor inhibitors exhibit varying degrees of effectiveness (111, 112), such as mTOR inhibitors (such as rapamycin), HIF-1 α inhibitors (such as px-478), and lipid metabolism regulators (such as ACC1 inhibitors). The development of specific nanodrugs or CAR-T cell therapies holds promise for enhancing targeted intraocular treatments (113), while cytokine-based therapies are emerging as a promising treatment option (114, 115), including strategies to jointly target metabolism and cytokines (such as anti-IL-17 mAb).

However, the clinical application of new small synthetic compounds and metabolic regulators requires extensive experimental validation, and challenges remain (105). Therefore, an interdisciplinary approach integrating immunology, metabolism, ophthalmology, and pharmacology will be crucial for advancing our understanding of Th17 cell metabolism in uveitis and for developing personalized treatment strategies.
